# Development of nanocomposite hydrogel using citrate-containing amorphous calcium phosphate and gelatin methacrylate

**DOI:** 10.3389/fbioe.2024.1421415

**Published:** 2024-09-10

**Authors:** Abhishek Indurkar, Kristaps Rubenis, Aldo R. Boccaccini, Janis Locs

**Affiliations:** ^1^ Institute of Biomaterials and Bioengineering, Faculty of Natural Sciences and Technology, Riga Technical University, Riga, Latvia; ^2^ Baltic Biomaterials Centre of Excellence, Headquarters at Riga Technical University, Riga, Latvia; ^3^ Department of Material Science and Engineering, Institute of Biomaterials, University of Erlangen-Nuremberg, Erlangen, Germany

**Keywords:** nanocomposite hydrogel, amorphous calcium phosphate, citrate-containing amorphous calcium phosphate, gelatin methacrylate, chemical crosslinking, biomaterials, bone tissue engineering

## Abstract

Nanocomposite hydrogels are suitable in bone tissue engineering due to their resemblance with the extracellular matrix, ability to match complex geometries, and ability to provide a framework for cell attachment and proliferation. The nanocomposite hydrogel comprises organic and inorganic counterparts. Gelatin methacrylate (GELMA) is an extensively used organic biomaterial in tissue engineering due to its excellent biocompatibility, biodegradability, and bioactivity. The photo-crosslinking of GELMA presents a challenge when aiming to create thicker nanocomposite hydrogels due to opacity induced by fillers, which obstructs the penetration of ultraviolet (UV) light. Therefore, using a chemical crosslinking approach, we have developed nanocomposite GELMA hydrogel in this study by incorporating citrate-containing amorphous calcium phosphate (ACP_CIT). Ammonium persulfate (APS) and Tetramethylethylenediamine (TEMED) were deployed to crosslink the methacrylate group of GELMA. The oscillatory shear tests have confirmed that crosslinking enhances both storage (G′) and loss modulus (G″) of GELMA. Subsequently, incorporation of ACP_CIT in GELMA hydrogel shows further enhancement in G′ and G″ values. *In vitro* analysis of the developed hydrogels revealed that chemical crosslinking and incorporation of ACP_CIT do not compromise the cytocompatibility of the GELMA. Hence, for developing nanocomposite GELMA hydrogels employing APS/TEMED crosslinking emerges as a promising alternative to photo-crosslinking.

## Graphical Abstract

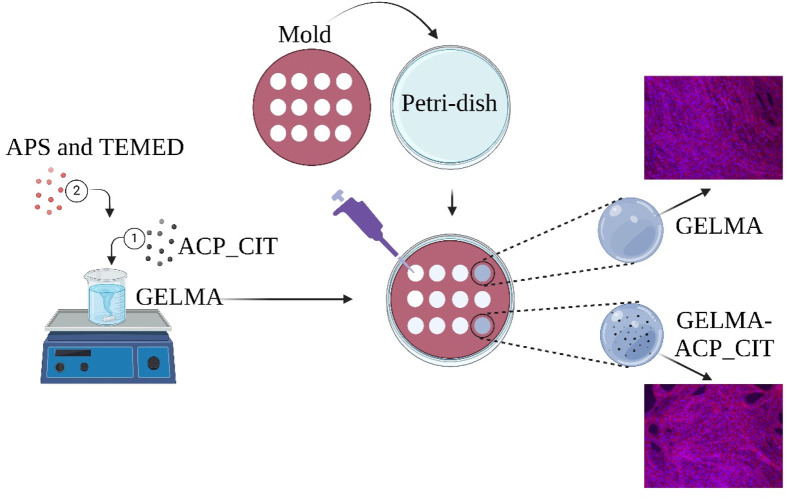

## 1 Introduction

The nanostructure arrangement of the extracellular matrix (ECM) provides appropriate physical and biological properties to bone. Nanocomposites are preferred for mimicking bone tissue as they provide an appropriate matrix environment and integrate desired biological properties (Sahoo et al., 2013). Nanocomposites contain organic (hydrogel) and inorganic (nanofiller) components (Indurkar et al., 2023a). The organic component is conducive to cell proliferation, nutrient, and waste transport, while the nanofiller enhances mechanical properties by hydrogen bonding, hydrophobic interaction, or charge interactions with the hydrogel (Phogat et al., 2023). Nanocomposite hydrogels provide superior reinforcement potential, biomolecule delivery, and tunable degradability compared to pure hydrogel (Nallusamy and Das, 2021). Therefore, it can be considered a preferred strategy for bone tissue regeneration.

GELMA is an attractive material in tissue engineering due to its biocompatibility, biodegradability, bioactivity, and unique crosslinking properties in developing nanocomposite hydrogels. The versatile bio-functionality of GELMA arises from the arginine-lysine-aspartic acid (RGD) motifs essential for cell attachment (González-Gamboa et al., 2022). A commonly used method for crosslinking GELMA hydrogels is photo-crosslinking, wherein ultraviolet (UV) light is used with photoinitiator, which enters a high-energy radical state and acts on the reactive functional group of GELMA (Zhang et al., 2023). The major shortcoming of photo-crosslinking is limited light penetration depth caused by decreased light intensity along the height of the hydrogel to be cured. A 5–200 μm layer or up to a few millimeters can be cured effectively. However, as the height increases, the light intensity decreases, decaying light intensity within the material according to the Beer-Lambert law. The photoinitiator absorbs the incident light at the top of the hydrogel, leading to a top-to-bottom crosslinking gradient (Klikovits et al., 2022). Moreover, filler addition deteriorates hydrogel’s transparency, further complicating the photo-crosslinking process. The incorporation of filler makes opaque hydrogels, which can reflect, refract, and absorb light, which decreases the intensity of penetration light (Gao et al., 2024).

A redox system that uses a chemical initiator (APS and TEMED) is relatively simple and effective in overcoming GELMA’s shortcomings in developing nanocomposite hydrogels. Like photoinitiated crosslinking, the APS/TEMED crosslinking approach works on free radical polymerization. The addition of TEMED accelerates the scission of ammonium persulfate (APS), forming disulfide radicles and hydroxyl radicles (Seetharaman et al., 2017). These free radicles snatch one electron from the carbon-carbon double bonds in the methacrylate group of the GELMA monomer and later become free radicles, which binds the monomers together to form long aliphatic chains that are consequently crosslinked (Tsanaktsidou et al., 2019). The crosslinking mechanism is reported in previous studies (Park et al., 2018).

Amorphous calcium phosphate (ACP) is the precursor of hydroxyapatite (HAP) and possesses remineralization potential (Bian et al., 2024). However, due to ACP’s metastability, it rapidly converts to HAP, resulting in a loss of remineralization activity (Yan et al., 2022). To prolong the remineralization capacity of ACP, stabilizing amorphous nature is of prime importance. Various organic and inorganic stabilizing agents have been used previously to prolong the crystallization of ACP (Chen et al., 2014). In previous studies, citrate was proven to stabilize ACP and delayed its transformation to HAP (Chatzipanagis et al., 2016; Schweikle et al., 2019; Degli Esposti et al., 2022; Indurkar et al., 2023c).

Given the metastable nature of ACP, a faster crosslinking process is required to maintain its remineralization potential, in contrast to the stable crystalline forms of calcium phosphate. Therefore, citrate-stabilized ACP (ACP_CIT) was synthesized and used as an inorganic filler in developing nanocomposite GELMA hydrogels. Rapid crosslinking was achieved using APS/TEMED while effectively addressing the limitation of photo-crosslinking. The impact of APS/TEMED crosslinking on the morphology of ACP_CIT and the effect of ACP_CIT on the rheological properties and cytocompatibility of GELMA hydrogel was also evaluated.

## 2 Materials and methods

### 2.1 Materials

Calcium citrate tetrahydrate (CAS 5785–44–4), trisodium phosphate (CAS 7601–54–9), sodium hydroxide (CAS 1301–73–2), ammonium persulfate (CAS 7727–54–0), and N, N, N′, N′-Tetramethylethylenediamine (CAS 110–18–9) were procured from Sigma Aldrich, Germany. Methacrylate gelatin (GELMA) with ∼50% degree of methacrylate was obtained from Cellink, Sweden. Hanks balanced salt solution (HBSS) was acquired from Gibco Life Technologies, Germany.

### 2.2 Synthesis of ACP_CIT

The synthesis of ACP_CIT was performed according to the previously reported procedure (Indurkar et al., 2023b). Briefly, the reaction was performed in a volume of 300 mL. Initially, 150 mL of 50 mM of calcium citrate solution was prepared in Milli-Q^®^ water, followed by pH adjustment to 11.5 using 2 M sodium hydroxide. Subsequently, 150 mL of 100 mM trisodium phosphate solution was added rapidly into 150 mL of 50 mM of calcium citrate solution. The precipitate was isolated by centrifuging at 3,000 rpm for 5 min, and the precipitate was washed thrice with Milli-Q^®^ water. Subsequently, the centrifuge tubes containing the precipitates were immersed in liquid nitrogen for 15 min, followed by freeze-drying for 72 h. The obtained powder was preserved in airtight containers for further characterization.

### 2.3 Characterization

The phase composition of synthesized ACP_CIT was determined using X-ray diffraction (XRD) and performed with a PANalytical Aeris diffractometer (Netherlands). The diffraction data were collected at 40 kV and 15 mA in a step mode with a step size of 0.04°, in the 2θ range from 10° to 70°.

Fourier-transformed infrared spectroscopy (FTIR) analysis was performed using Nicolet iS50 FT-IR spectrometer (Thermo Scientific, Waltham, MA, United States). Experiments were performed in transmission mode from the wavenumber ranging from 4,000 to 400 cm^-1^ with a resolution of 4 cm^-1^ (64 scans).

The morphology and particle size of ACP_CIT were evaluated by Field Emission Gun Transmission Electron Microscopy (FEG-TEM, Tecnai G2 F30, United States) operated at 300 kV. The sample preparation was as follows: a small amount of ACP-CIT powder was dispersed in isopropyl alcohol and sonicated in an ultrasonic bath. Further, the samples were placed on a carbon-coated grid and dried before analysis.

### 2.4 Synthesis of the hydrogels

Two sets of hydrogels were prepared: GELMA and nanocomposite GELMA-ACP_CIT. The GELMA hydrogel was prepared by dissolving 5 mL of (5% w/v) GELMA in Milli-Q^®^ water at 50°C. Subsequently, the nanocomposite hydrogel was prepared by adding 100 mg of ACP_CIT (2% w/v) in 5 mL of (5% w/v) GELMA solution and stirred for 10 min at 500 rpm. Subsequently, 100 μL of APS (10% w/v) and 10 μL TEMED were added to GELMA and GELMA-ACP_CIT solution. After mixing, each hydrogel was poured into the 12 mm diameter and 2 mm height silicon molds using a displacement pipette. The hydrogels were allowed to crosslink for 30 min at 37°C.

### 2.5 Oscillatory shear tests

Oscillatory shear tests were performed with a TA HR20 rheometer (United States) to examine the hydrogels’ viscoelastic properties. A 20 mm diameter parallel plate geometry with a solvent trap was used. Each sample was analyzed in triplicate, presenting average and standard deviation data. The linear viscoelastic region (LVE) was analyzed by amplitude sweep analysis, and complex viscosity was performed under a constant frequency of 1 Hz and shear stress ranging from 1% to 1,000%. The loss moduli (G″) and storage moduli (G′) were characterized by the frequency sweep analysis performed under 1% strain under the frequency range from 0.1 to 10 Hz. All the analyses were performed at a constant temperature of 37°C in triplicate, and data is presented as average with standard deviation.

### 2.6 Field emission scanning electron microscopy

The micro and nanostructure of samples were examined using a high-resolution field emission scanning electron microscope SEM/STEM (Verios 5 UC, Thermo Fisher). SEM images were acquired utilizing through-lens (TLD) and Everhart-Thornley (ETD) detectors, operated at 2 kV. The samples were coated with a 10 nm carbon layer using a LEICA EM ACE 200 coater. A low current and vector scanning approach with a dwell time of 50 ns was employed to prevent charging and sample damage. Samples were transferred onto LacyCarbon support film grids for STEM measurements. Images were captured using a BF STEM3 detector, operated at 30 kV.

### 2.7 *In vitro* analysis

#### 2.7.1 Cell culture maintenance

An osteoblast precursor cell line derived from mouse (*Mus musculus*) calvaria (MC3T3-E1, Sigma Aldrich, Germany) was employed for cellular analysis after ten passages. MC3T3-E1 cells were maintained in an α-MEM medium containing 10vol% Fetal bovine serum (Gibco Life Science, United States) and 1vol% penicillium-streptomycin (Thermo Fisher Scientific, Waltham, MA, United States) at 37°C in a humidified atmosphere of 95% air and 5% CO_2_. The cultures of MC3T3-E1 cells were trypsinized by adding 3 mL of trypsin-EDTA solution. When the cells were detached, 9 mL of α-MEM medium was added to the T75 flask. The cells were counted, and 1 × 10^5^ cells/mL were inoculated into fresh T-75 flasks, followed by incubation at 37°C in a humidified atmosphere of 95% air and 5% CO_2_.

#### 2.7.2 Cell harvesting

Cell culture media was removed from the T-75 flask, followed by adding 5 mL of sterile Dulbecco’s phosphate buffer saline (DPBS) (Thermo Fisher Scientific, Waltham, MA, United States) to dislodge loosely attached cells and remove fractions. Then, the DPBS was discarded, adding 3 mL of 0.25% Trypsin-EDTA (Thermo Fisher Scientific, Waltham, MA, United States) solution for 3–5 min and incubating at 37°C for 3–5 min. After detachment of cells, 9 mL of α-MEM medium was added and mixed well and then transferred to a centrifuge tube at 350 rpm for 2 min to acquire cell pellet, followed by redispersion in 3 mL of cell medium and mixed well. 100 μL of the cell suspension was transferred to 96-well plates, later 100 μL of trypan blue was added, and cells were further counted using a Neubauer chamber (Neubauer-improved, Paul Marienfeld Gmbh and Co.Kg, Germany). A solution containing 25,000 cells per ml was prepared and centrifuged at 350 rpm for 2 min to acquire a cell pellet.

#### 2.7.3 Cell encapsulation

For the cellular analysis, sterile synthesis of ACP_CIT and GELMA was performed. All the reagents were passed through 0.22 μm Millipore filters, whereas calcium citrate and Milli-Q^®^ water were autoclaved at 121°C for 15 psi for 30 min. The obtained cell pellet (Section 2.7.2) was mixed in 5% (w/v) GELMA solutions and crosslinked with sterile filtered 100 μL of APS (10% w/v) and 10 μL TEMED. The hydrogel was poured into the silicon molds and allowed to crosslink for 30 min at room temperature. The same procedure was performed for encapsulation in ACP_CIT-GELMA hydrogels. The hydrogels were incubated in the α-MEM medium at 37°C in a humidified atmosphere of 95% air and 5% CO_2_. The cell culture analysis was performed for 7 days, and the medium was changed every 2 days.

#### 2.7.4 Rhodamine phalloidin/DAPI staining

Cell morphology in hydrogels was examined under a fluorescence microscope (Axio, Carl Zeiss, Jena, Germany). Samples (n = 3) were treated with 4% formaldehyde solution for 5 min in the dark. Subsequently, the samples were treated with 0.1% Triton-X solution for permeation. Further, the samples were washed twice with HBSS. Afterward, the samples were stained with 5 μL/mL solution of Rhodamine-Phalloidin staining (Thermofisher Scientific, United States) for 1 h followed by 1 μL/mL DAPI (Thermofisher Scientific, United States) solution for 5 min in the dark. Further, the samples were washed, immersed in HBSS solution, and analyzed under a fluorescence microscope (Primo Vert Axio, Zeiss, Oberkochen, Germany).

## 3 Result and discussion

### 3.1 Characterization

**FIGURE 1:**
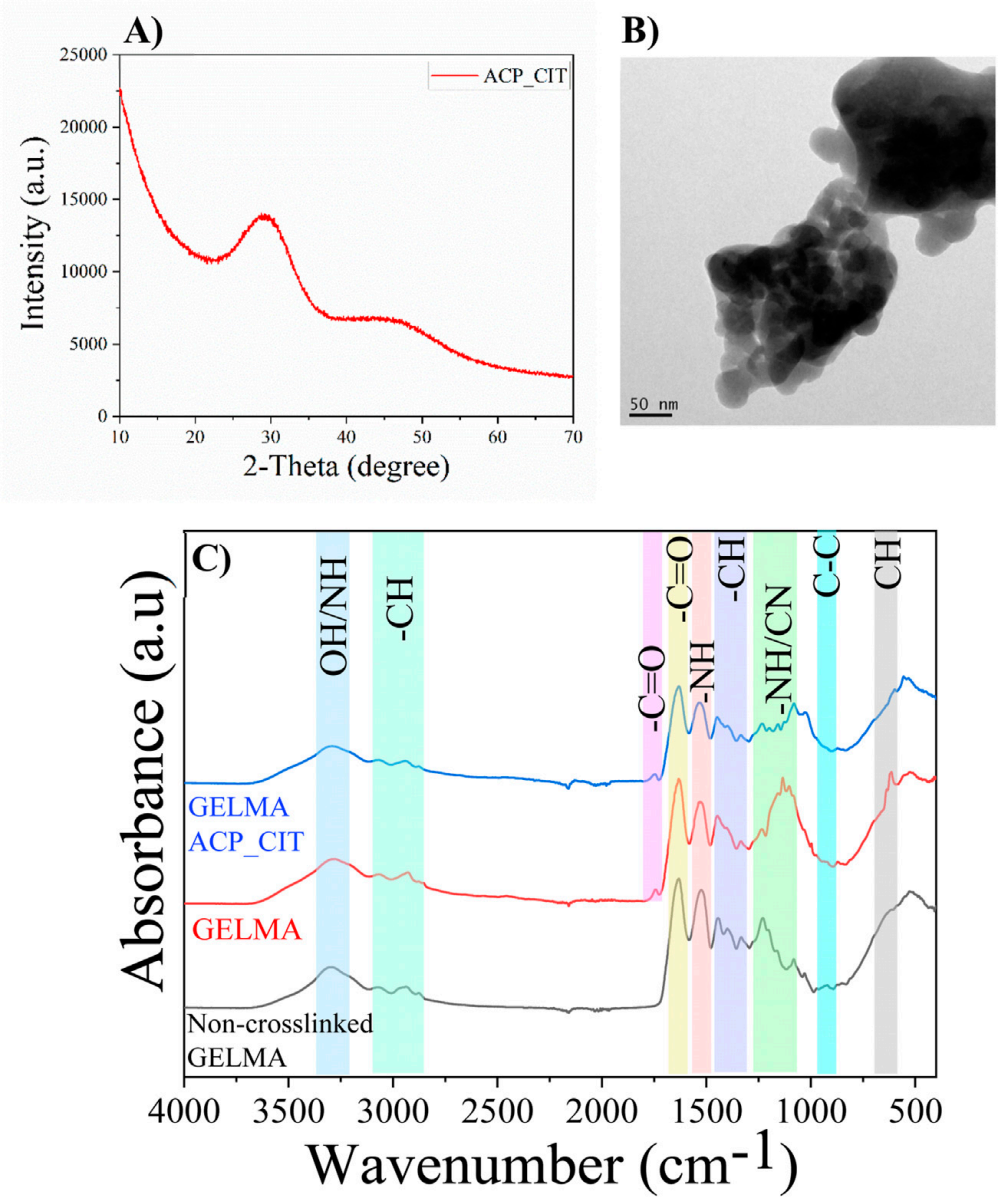
XRD **(A)**, the morphology of synthesized ACP_CIT **(B)**, and FTIR of Non-crosslinked GELMA, radically GELMA (non-crosslinked and crosslinked), and GELMA-ACP_CIT nanocomposite hydrogel **(C)**.

The lack of crystalline order confirms the formation of ACP_CIT, as shown in Figure 1A. Detailed characterization of ACP_CIT was reported in our previous publication (Indurkar et al., 2023b). The FEG-TEM analysis in Figure 1B reveals the spherical morphology and particle size of ∼40 nm of synthesized ACP_CIT.

The FTIR analysis of non-crosslinked GELMA and APS/TEMED crosslinked hydrogels (GELMA and GELMA-ACP_CIT) are shown in Figure 1C. The characteristic peaks of O-H and N-H group stretching vibrations were observed at 3,300 cm^-1^. The peaks in the regions 3,100–2,800 cm^-1^ correspond to the CH_2_ stretching vibrations. The characteristics of amide bands of gelatin were observed around i) 1,632 cm^-1^ representing C=O stretching of amide I, ii) 1,533 cm^-1^ representing N-H bending coupled with C-H stretching of amide II, iii) 1,232 cm^-1^ was denoted to C-N stretching and N-H bending of amide III, iv) 922 cm^-1^ indicating -C-C- the skeletal stretch of amide IV and v) 615 cm^-1^ corresponds to the CH out-off plane skeletal stretch of amide V (Elsayed et al., 2018). The results indicate that all the amide bands of gelatin remain intact after crosslinking. It is worth noting that the characteristic peak around 1,640 cm^-1^ in the GELMA spectrum corresponds to the C=C stretching of the methacrylate group, which is too close to C=O amide-I stretching. Therefore, it is difficult to distinguish and verify the disappearance of C=C after crosslinking (Fonseca et al., 2020).

### 3.2 Hydrogel characterization

**FIGURE 2:**
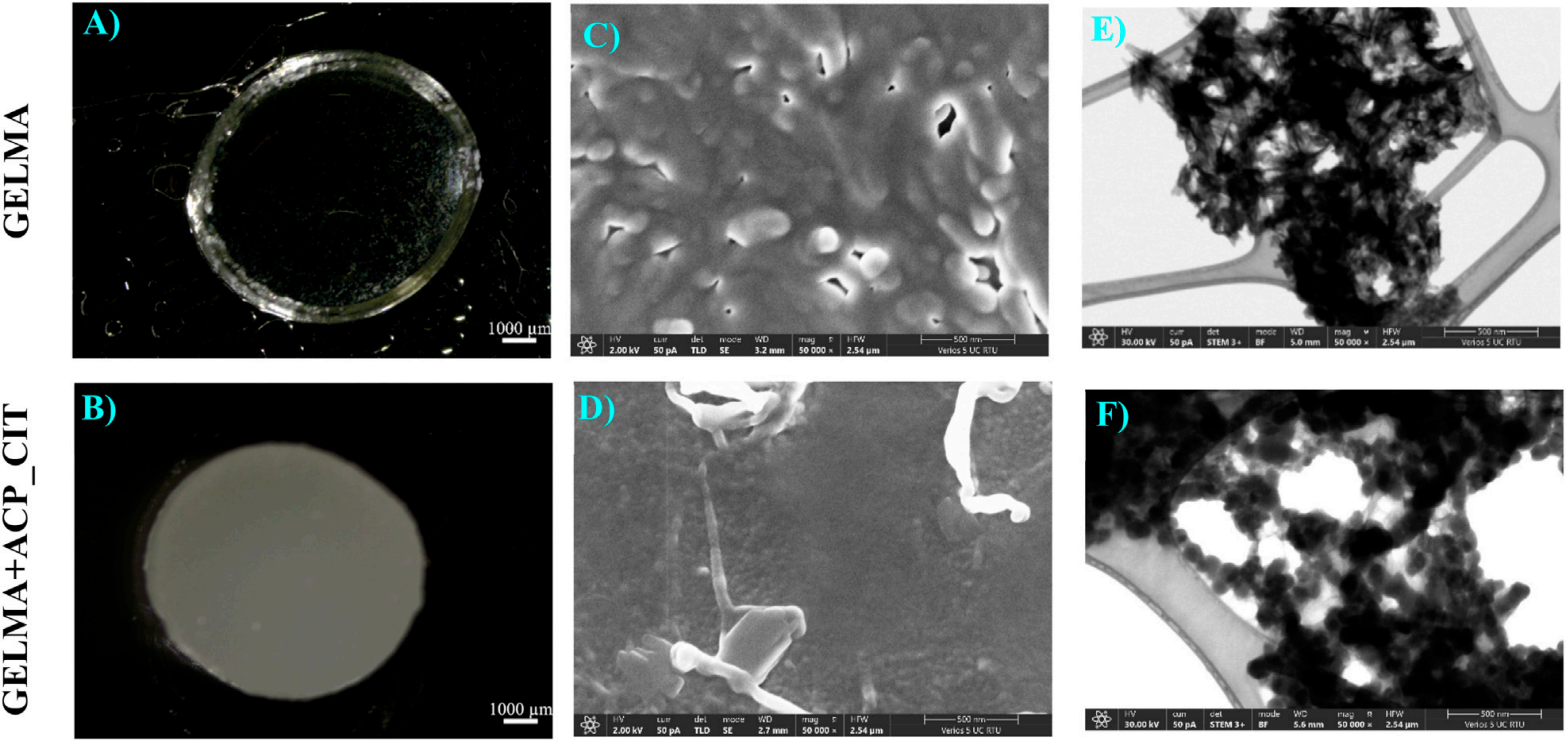
APS/TEMED crosslinked GELMA **(A)** and nanocomposite GELMA scaffolds containing ACP_CIT **(B)**. Micro and nanostructure analysis of APS/TEMED crosslinked GELMA **(C–E)** and nanocomposite GELMA scaffolds containing ACP_CIT **(D–F)**.

The synthesized hydrogels are shown in Figures 2A, B. The GELMA forms transparent hydrogels, therefore making it suitable for photo-crosslinking. However, the incorporation of ACP_CIT makes GELMA hydrogels opaque.

Recent studies have revealed that the opacity of the hydrogel obstructs the photo-crosslinking potential of GELMA. Nanocomposites GELMA hydrogel containing nanohydroxyapatite (nHA) revealed incomplete crosslinking of GELMA/nHA inside a multilayer scaffold (Liu et al., 2019). Similarly, incorporating amorphous magnesium phosphate in GELMA leads to opaque hydrogels, making crosslinking difficult and affecting the stiffness of the hydrogel (Dubey et al., 2020). Crosslinking is essential to maintain the structural integrity of hydrogel or scaffold. Due to the limitation of photo-crosslinking, there is a pressing need to develop a chemical crosslinking approach for creating nanocomposite GELMA hydrogel. The APS/TEMED chemical crosslinking is a simple approach that results in quick crosslinking of nanocomposite GELMA hydrogel containing ACP_CIT, as shown in Figure 2B.

The surface microstructure of APS/TEMED crosslinked GELMA and nanocomposite GELMA scaffolds containing ACP_CIT is shown in Figures 2C, D. The nanostructure of the hydrogel was examining the morphology of the ACP_CIT. The ACP_CIT nanoparticles maintain their spherical morphology and are well embedded into the GELMA matrix, as shown in Figure 2F. The structural analysis has indicated that the APS/TEMED crosslinking does not hamper the spherical morphology of ACP_CIT.

### 3.3 Oscillatory shear tests

**FIGURE 3:**
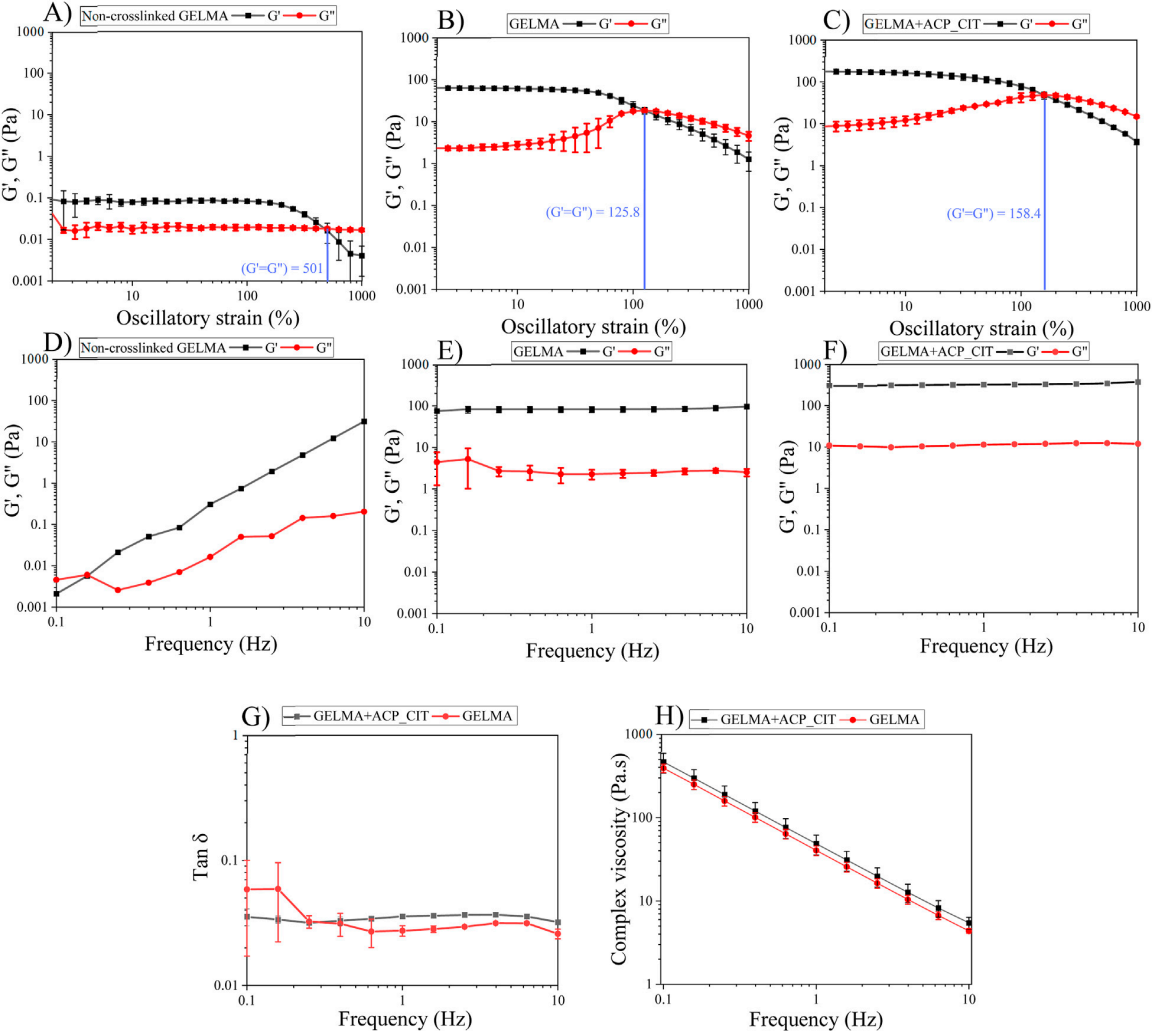
The viscoelastic properties of the hydrogels were analyzed using oscillatory shear tests: the LVE region of the hydrogel was analyzed by amplitude sweep **(A–C)**. Investigation of storage and loss modulus of the hydrogels by frequency sweep **(D–F)**. Tan δ analysis **(G)**, and complex viscosity test analyzing the flow properties of the hydrogels **(H)**.

Analyzing the linear viscoelastic region of the hydrogels (LVE) is the first step in evaluating the viscoelastic properties shown in Figures 3A–C. The storage modulus (G′) represents the elastic property of viscoelastic material, indicating the stored deformation energy. In contrast, the loss modulus (G″) corresponds to the viscous property of viscoelastic, indicating deformation energy lost through internal friction when flowing. When G′>G″, the material possesses viscoelastic solid-like properties. On the other hand, when G′<G″ the material possesses viscoelastic fluid-like behavior (Indurkar et al., 2020). The GELMA (non-crosslinked), GELMA and GELMA-ACP_CIT exhibits G′>G″ corresponding to viscoelastic gel-like behavior with LVE region falling within 10% of strain. On increasing the strain, the crossover point is reached where G′ = G″. After the crossover point, the three-dimensional hydrogel network completely ruptures (Pereira et al., 2022).

The polymeric chain in the non-crosslinked hydrogel is free and exhibits a high degree of freedom. Therefore, the ability to withstand irreversible strain-induced deformation is high, so the crossover point of GELMA was observed at 501% strain. However, after crosslinking with APS/TEMED, the polymeric chains are linked together, observed from enhancement in G′ and G″; therefore, the ability to withstand irreversible strain-induced deformation is reduced. Incorporating ACP_CIT in GELMA hydrogel enhances the crossover point to 158%, imparting the ability to withstand higher stains.

The frequency sweep analysis was performed at a constant strain of 1% within the LVE region. The G′ and G″ were analyzed against frequency, as shown in Figures 3D–F. At lower frequencies, the G″ of non-crosslinked GELMA hydrogel dominates over the G′ value corresponding to the sol-gel behavior. With increased frequency, the G′ drastically increases and takes over G″ values. Conversely, the G′ and G″ of GELMA and GELMA-ACP_CIT were independent of frequency.

The G′ and G″ of each sample were analyzed at 1 Hz frequency. The non-crosslinked GELMA hydrogel has very low G′ of 0.12 Pa and G″ of 0.016 Pa, indicating its fragile nature (Dulong et al., 2024). The moduli of the GELMA hydrogel were found to be frequency-dependent. G″ dominates G′ at lower frequencies while at higher frequencies G′ exceeds G″ reflecting its viscoelastic liquid-like behavior (Figure 3D). Upon crosslinking, the moduli of both GELMA and GELMA-ACP_CIT become independent of frequency exhibiting viscoelastic gel-like properties (Ramli et al., 2022). The crosslinking significantly enhanced the moduli of the GELMA, with G′ increasing to 82.58 ± 13 Pa and G″ of 2.25 ± 0.5 Pa (Figure 3E). Furthermore, incorporation of ACP_CIT in GELMA has resulted in further increase with G′ reaching to 318.8 ± 6.5 Pa and G″ of 11.3 ± 0.2 Pa, thus confirming the reinforcement effect (Figure 3F). The moduli of GELMA and GELMA-ACP_CIT were independent of frequency; therefore, further analysis was performed on these two samples.

Tan δ is the G″ to G′ ratio often used to analyze the damping factor of the hydrogels represented in Figure 3G. When the tan δ > 1 sample behaves like a viscous liquid, the tan δ < 1 sample behaves more like an elastic solid (Yan and Pochan, 2010). At 1 Hz, the tan δ of GELMA was 0.035 ± 0.0006. On incorporating ACP_CIT, the polymer movement may have been confined, thus decreasing the tan δ (0.027 ± 0.002) of GELMA-ACP_CIT (SudarshanRao, 2021).

Shear-thinning or pseudoplastic flow behavior is essential to pressure or mechanically assisted extrusion-based biofabrication systems. For example, during extrusion, the hydrogel or bioink undergoes stress through a small orifice, adversely affecting the viability of the suspended cells and the shape fidelity of the printed construct. However, shear-thinning properties reduce the shear stress, diminishing the risk of cell death and maintaining the printed construct’s pattern fidelity (Moon et al., 2024). The flow behavior of hydrogel shown in Figure 3H demonstrates the shear-thinning behavior of the GELMA and GELMA-ACP_CIT hydrogels (Vlachopoulos and Strutt, 2016). Therefore, these hydrogels can effectively be utilized as biomaterials for pressure or mechanically assisted extrusion-based biofabrication systems.

### 3.4 Rhodamine phalloidin and DAPI staining

**FIGURE 4:**
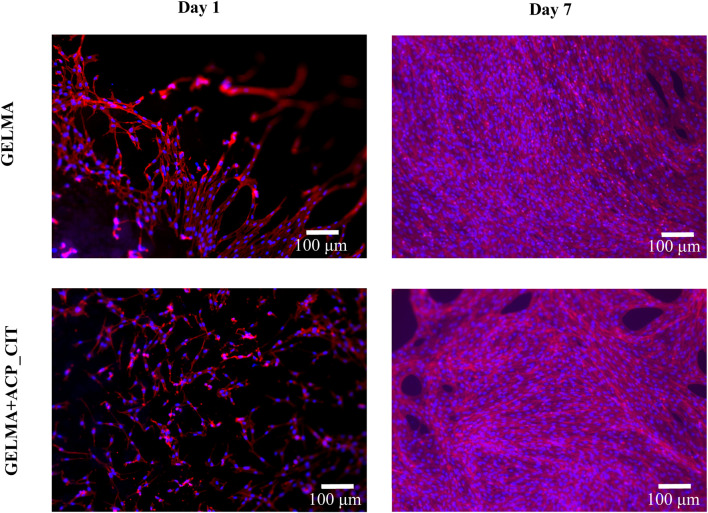
Fluorescent microscopy of rhodamine-phalloidin (red) and DAPI (blue) staining of MC3T3-E1 cells embedded in GELMA and GELMA-ACP_CIT hydrogels.

*In vitro* analysis evaluated the effect of the APS/TEMED crosslinking and ACP_CIT incorporation in GELMA hydrogel. The fluorescent microscopy images are presented in Figure 4, which show the cytoskeleton (red) and the nucleus (blue) of MC3T3-E1 cells. Cell attachment was observed on the first day, and by the seventh day, the cells were spread and distributed well on the hydrogel, confirming the cytocompatibility of APS/TEMED crosslinking. The preliminary analysis indicates that APS/TEMED crosslinking and incorporation of ACP_CIT have not shown adverse effects on the cytocompatibility of GELMA.

## 4 Conclusion

The nanocomposite hydrogels of GELMA containing ACP_CIT were developed successfully using chemical crosslinking. The FTIR analysis revealed that the APS/TEMED crosslinking did not alter the functional groups of GELMA. Subsequently, the oscillatory shear tests revealed that the non-crosslinked GELMA possesses very low G′ and G″ values (less than 0.1 Pa). On crosslinking, G′ (82.58 ± 13 Pa) and G″ (2.25 ± 0.5 Pa) were significantly enhanced imparting structural integrity to the scaffold. On incorporation of ACP_CIT in GELMA, a further enhancement in G′ (318.8 ± 6.5 Pa) and G” (11.3 ± 0.2 Pa) confirms the reinforcement potential. Finally, the *in vitro* analysis has concluded that APS/TEMED and ACP_CIT do not obstruct the growth of MC3T3-E1 cells. The preliminary analysis has shown the cytocompatibility of chemical crosslinking and ACP_CIT incorporation in GELMA hydrogel. Therefore, the APS/TEMED crosslinking can be further explored in various processing approaches (bioprinting, electrospinning, electro-spraying, freeze-dyring) to develop GELMA-based nanocomposites.

## Data Availability

The raw data supporting the conclusions of this article will be made available by the authors, without undue reservation.
